# A novel extramedullary technique to guide femoral bone preparation in mobile unicompartmental knee arthroplasty based on tibial cut and overall alignment

**DOI:** 10.1186/s13018-020-01598-6

**Published:** 2020-03-05

**Authors:** Qidong Zhang, Weiguo Wang, Zhaohui Liu, Debo Yue, Liming Cheng, Bailiang Wang, Wanshou Guo

**Affiliations:** Department of Orthopaedic Surgery, China-Japan Friendship Hospital, Peking Union Medical College, Beijing, 100029 China

**Keywords:** Unicompartmental knee arthroplasty, Surgical technique, Extramedullary, Radiologic, Clinical outcome

## Abstract

**Background:**

The mobile Oxford unicompartmental knee arthroplasty (UKA) implant has been widely used with an intramedullary guide for femoral preparation. We modified the femoral guide technique based on the tibial cut first and spacer block technique. This study was performed to determine the radiographic accuracy and early clinical outcomes of the extramedullary method.

**Methods:**

We retrospectively evaluated 50 consecutive patients who underwent UKA using the extramedullary technique. An equal number of patients who underwent UKA with the conventional technique were matched as the control group. Clinical outcomes were evaluated in terms of the operating time, blood loss, range of motion, and Hospital for Special Surgery score. Radiographic accuracy was evaluated by the implant position and alignment in the coronal and sagittal planes.

**Results:**

The mean follow-up period was 39.76 ± 5.77 months. There were no differences in the postoperative Hospital for Special Surgery score, range of motion, or hip-knee-ankle angle between the two groups. The operating time in the extramedullary group was shorter than that in the conventional group (54.78 ± 7.95 vs. 59.14 ± 10.91 min, respectively; *p =* 0.025). The drop in hemoglobin after 3 days was only 12.34 ± 4.98 g/L in the extramedullary group which was less than that in the conventional group (*p =* 0.001). No significant differences were found in the postoperative coronal and sagittal angles between the two groups. Acceptable radiographic accuracy of the implant alignment and position was achieved in 92% of patients in the extramedullary group and 96% of patients in the conventional group.

**Conclusions:**

The radiographic and clinical results of the extramedullary technique were comparable with those of the conventional technique with the advantage of no intramedullary interruption, less blood loss, a shorter operating time, and more rapid recovery. As the technique depends on the accurate tibial cut and overall alignment, we do not recommend it to surgeons without high volume experiences.

**Trial registration:**

Retrospectively registered

**Level of evidence:**

IV, retrospective study

## Background

Unicompartmental knee arthroplasty (UKA) is a promising treatment option for osteoarthritis of the medial compartment of the knee because of its many advantages, such as a smaller incision, less soft tissue injury, and more rapid recovery [1–5]. The mobile Oxford UKA implant has been widely used with an intramedullary guide for standardized femoral preparation [6, 7]. However, this procedure is recognized as a technical challenge, especially when the device is implanted with a minimally invasive incision, which potentially results in component malposition and incorrect alignment [8]. Malposition increases the risk of bearing dislocation in mobile UKA, a serious complication that usually requires revision surgery [9–11]. Incorrect alignment in the coronal plane can also influence the outcome of UKA [12, 13]. Overcorrection causes contralateral compartmental overload and failure in arthritis progression. Undercorrection can increase the load to the medial compartment, which may accelerate polyethylene wear [14, 15]. Moreover, errors of alignment may cause changes in kinematics and implant loosening or failure [16–18].

In clinical practice, use of an intramedullary guide for standardized femoral preparation does not always guarantee accuracy of implant positioning and alignment. Additionally, the use of an intramedullary femoral guide has been associated with increased risks of blood loss, fat embolism, intraoperative fractures, and postoperative hypoxia [19]. An extramedullary femoral guide is used in total knee arthroplasty (TKA) systems. Many studies have compared the extramedullary femoral guide system with the conventional intramedullary guide system in TKA [20–23]. An extramedullary instrumentation system is also available in UKA. However, it is a fixed bearing system (Miller Galante unicompartmental prosthesis; Zimmer, Warsaw, IN, USA), which is designed quite differently from a mobile bearing system [24, 25]. The mobile Oxford UKA implant is designed with a congruous meniscal bearing, and no soft tissue release is performed. We found no reports of the extramedullary technique with use of the Oxford UKA implant after a thorough literature search.

We recently modified the surgical technique for guiding femoral bone preparation based on the tibial cut and overall alignment to perform UKA more easily and reproducibly without intramedullary interruption. The purpose of this study was to determine the accuracy of implant alignment and positioning using the extramedullary technique and to clarify the value of this extramedullary technique in mobile bearing UKA. We hypothesized that the outcome of the extramedullary technique would be substantially the same as that of the conventional technique, or at least not worse, without irritation of the intramedullary canal.

## Methods

The present study was approved by the institutional review board (No. 2013-SF-1). From May 2015 to January 2017, 50 consecutive knees were treated with UKA using the extramedullary technique. To compare the clinical outcomes, an equal number of knees that underwent UKA performed with the conventional technique during the same period was selected and matched as controls with respect to diagnosis (identical), age (± 3 years), preoperative range of motion (ROM) (± 5°), and radiological grade of knee arthrosis (identical). The indications for UKA were severe knee pain involving the medial compartment and considerable difficulty in walking and performing daily activities. Radiographs demonstrated medial loss of articular cartilage as evidenced by a narrow medial joint width. The other indications were an intact anterior cruciate ligament, varus deformity of < 15°, flexion contracture of < 15°, and an intact lateral compartment [26]. The preoperative diagnosis was osteoarthritis in all patients. Informed consent was obtained from all individual participants included in the study.

### Surgical procedure

All UKA procedures were performed by the senior surgeon with the mobile Oxford medial UKA device (Oxford unicompartmental knee; Biomet, Bridgend, UK). The knee joint was exposed through a small skin incision with quadriceps sparing and no patellar eversion. Medial release for ligament balancing was not performed. The tibial cut was performed first, allowing for subsequent femur preparation and gap control. The tibia cut was performed with an extramedullary guide. The shaft of the tibial cut guide was set parallel to the bone shaft. The proposed cut level was confirmed to be 2 to 3 mm below the deepest area of the erosion to prepare the baseplate. After performance of the tibial sagittal cut and transverse cut, the resected tibial bone was removed. The flexion gap was checked with a 7-mm gap gauge, which was mainly used to ensure that an appropriate amount of bone had been resected and that the tibial axial alignment was correct. This step was very important for the subsequent steps.

### Modified femoral preparation (see animation video in Additional file 1)

**Fig. 1:**
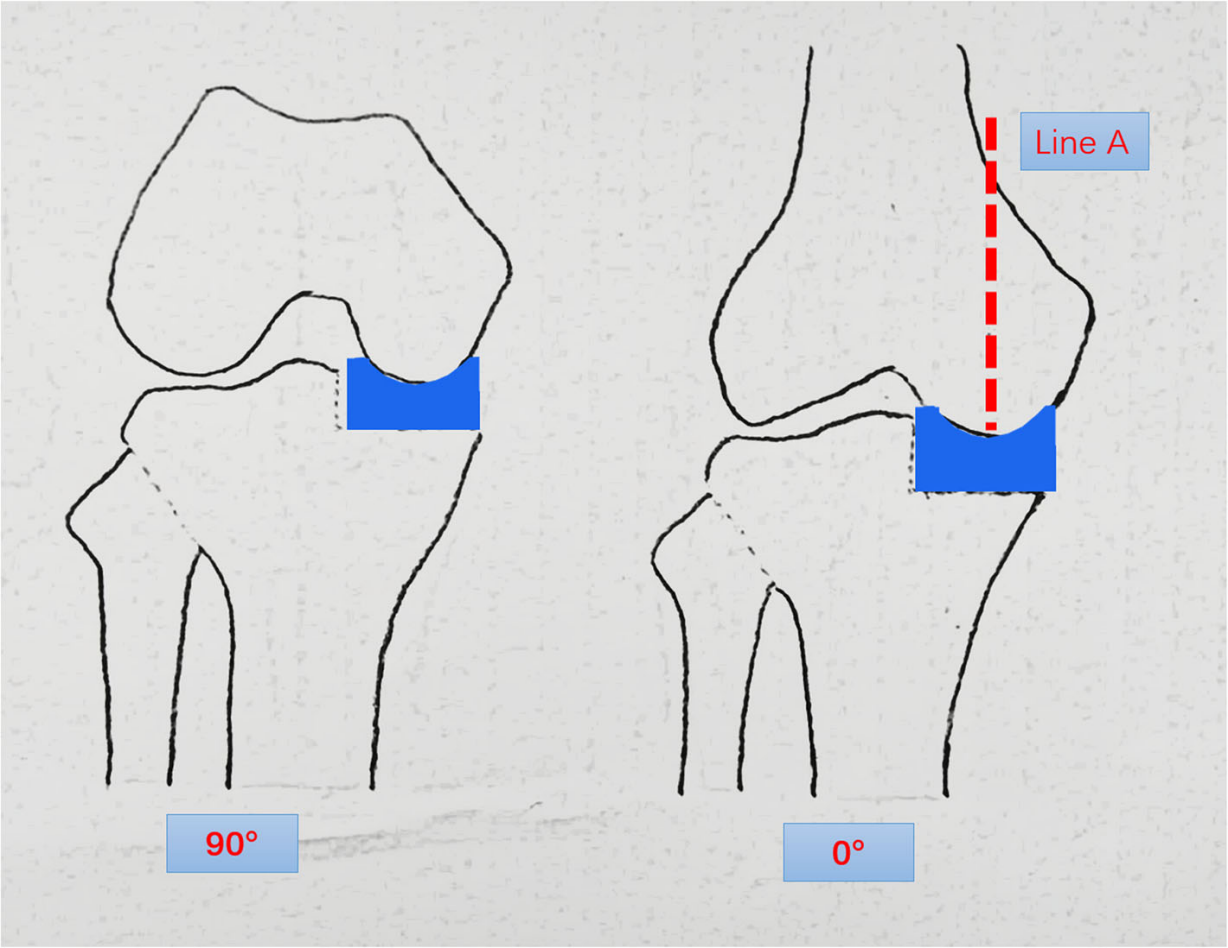
Following performance of the accurate tibial cut in flexion, the knee was brought into full extension. With an appropriate gap gauge (spacer block) in place, limb alignment was corrected to neutral. The femoral drill reference (line A) was drawn on the anterior femoral surface vertical to the tibial cut plane

**Fig. 2:**
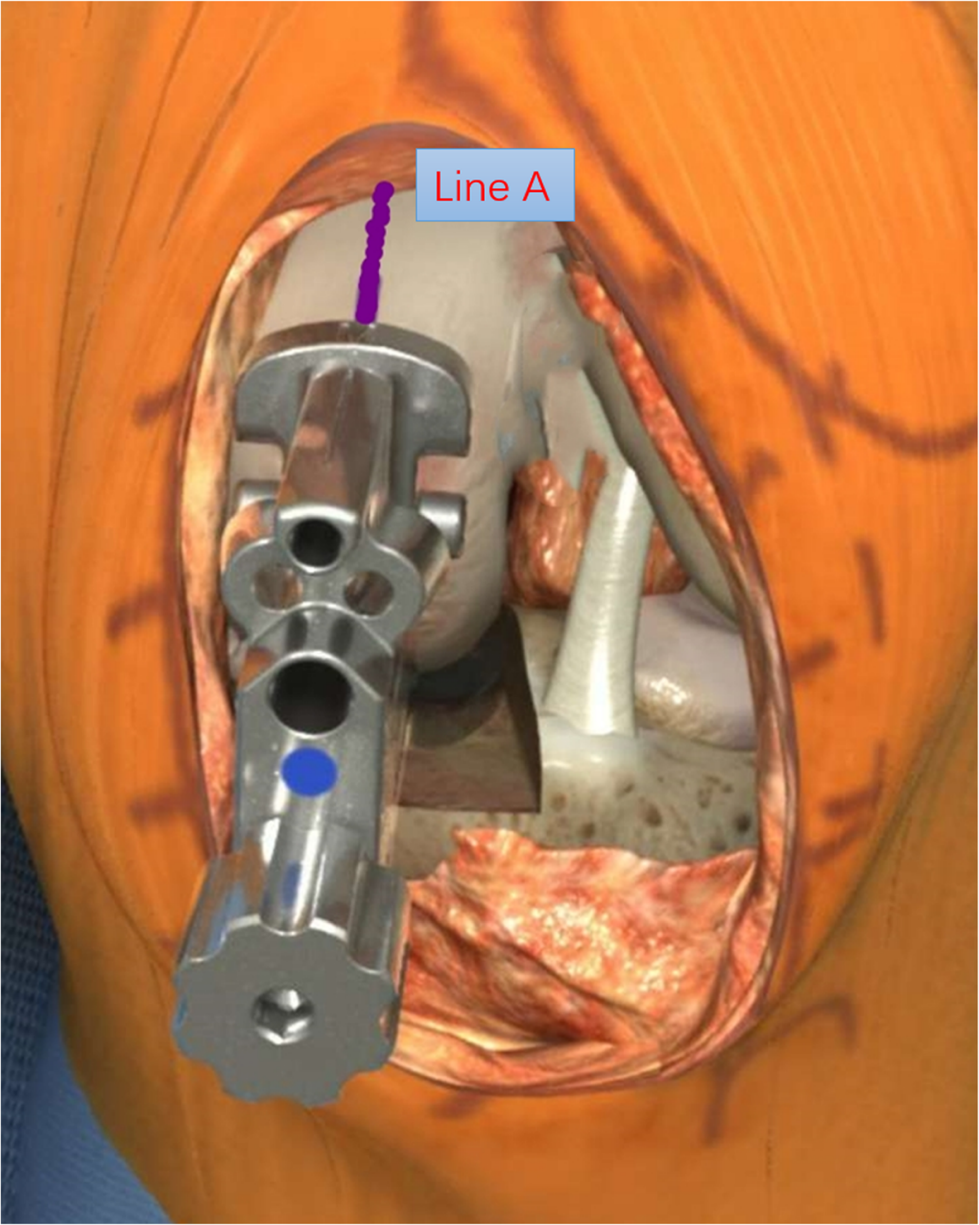
The femoral drill guide was visually aligned vertical to the tibial cut plane in the middle of the condyle and parallel to the femoral reference line (line A)

**Fig. 3:**
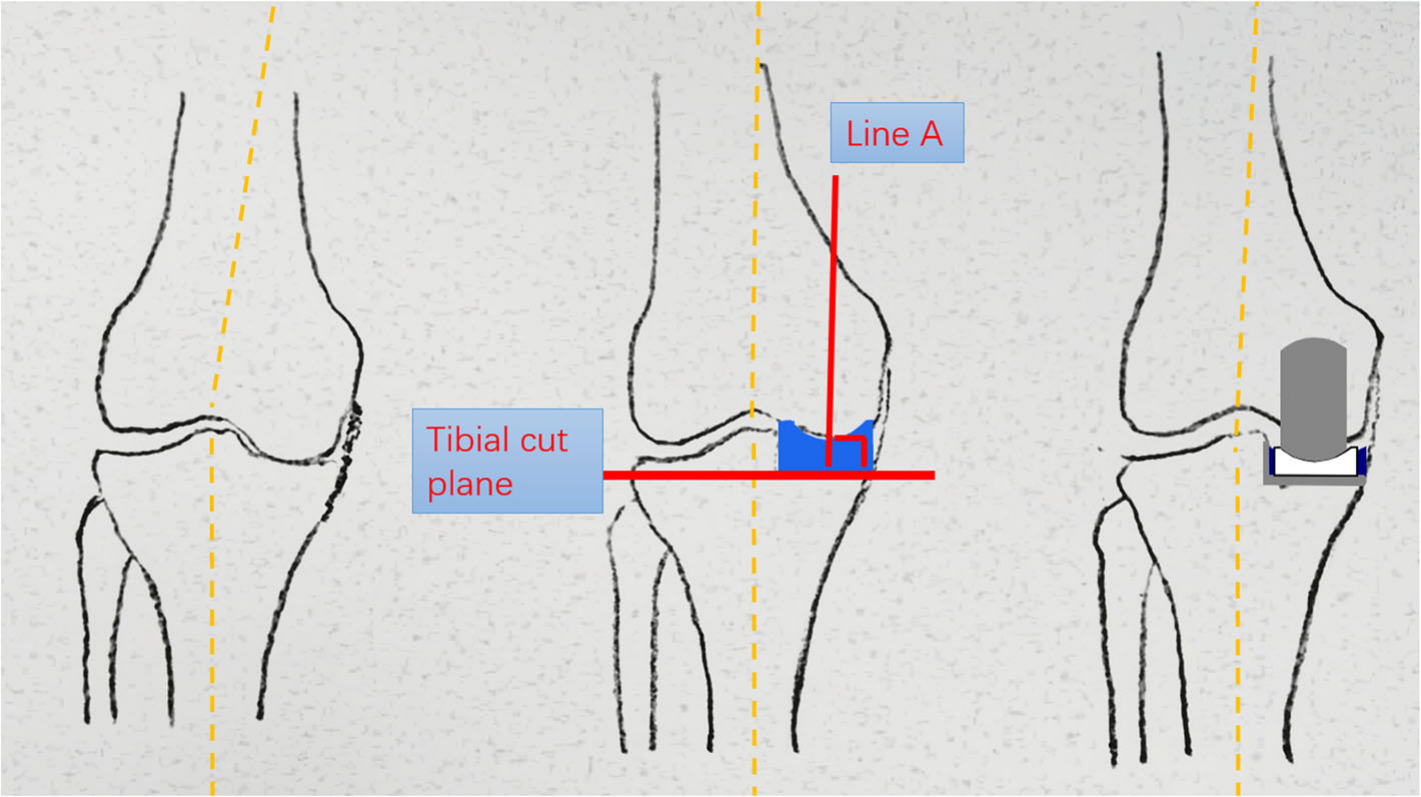
The principle of Oxford UKA is to maintain knee stability by ligament tension. The varus deformity may be due to medial wear, which is generally reducible during the operation. After the tibial cut, the knee was brought into full extension. With an appropriate gap gauge (spacer block) in place, the alignment was corrected to neutral by maintaining natural ligament tension. The femoral drill reference line (line A) was drawn on the femoral surface vertical to the tibial cut plane. Following the reference, femoral bone preparation was performed and the component was implanted. UKA thus achieved ideal alignment and implant positioning


Following performance of a precise tibial cut, the knee was brought into full extension and an 8-mm gap gauge (mainly used as a spacer block) was inserted into the joint space to correct varus deformity to neutral limb alignment by maintaining natural tension of the ligament because 1 to 2 mm of the distal femoral cartilage was lost and the posterior femoral cartilage was intact. If the 8-mm gap gauge was too loose, then a thicker gap gauge was inserted to correct the limb alignment by maintaining the natural ligament tension. With an appropriate gap gauge in place, a femoral drill alignment line (line A) was drawn on the anterior femoral surface vertical to the tibial cut plane. This line was an extremely important reference and was checked many times. The knee was flexed to 90°, and the femoral drill guide was inserted while fully attached to the distal femur and fully seated on the tibial cut surface. There were two alignment requirements for the femoral drill guide: The femoral drill direction must be parallel to line A, and the femoral drill guide must lie in the center of the medial condyle. Next, 4-mm and 6-mm drills were passed through the holes in the guide. The posterior femoral condyle was resected with a 4-mm femoral saw block, and the distal femoral condyle was then milled to balance the flexion gap and extension gap. The inserted polyethylene bearing should restore normal ligament tension without impingement or instability. Cement was used to fix the components (Figs. 1, 2, and 3).

**Additional file 1** Animation video.


### Clinical and radiographic assessments

The patients were followed up at 3, 6, and 12 months postoperatively and yearly thereafter. Clinical outcomes were evaluated in terms of the operating time, blood loss, knee Hospital for Special Surgery (HSS) score, ROM, and complications. Any complications such as fat embolism, deep venous thrombosis, fracture, infection, arthritis of lateral compartment, or loosening were recorded. The final assessment was recorded for analysis.

**Fig. 4:**
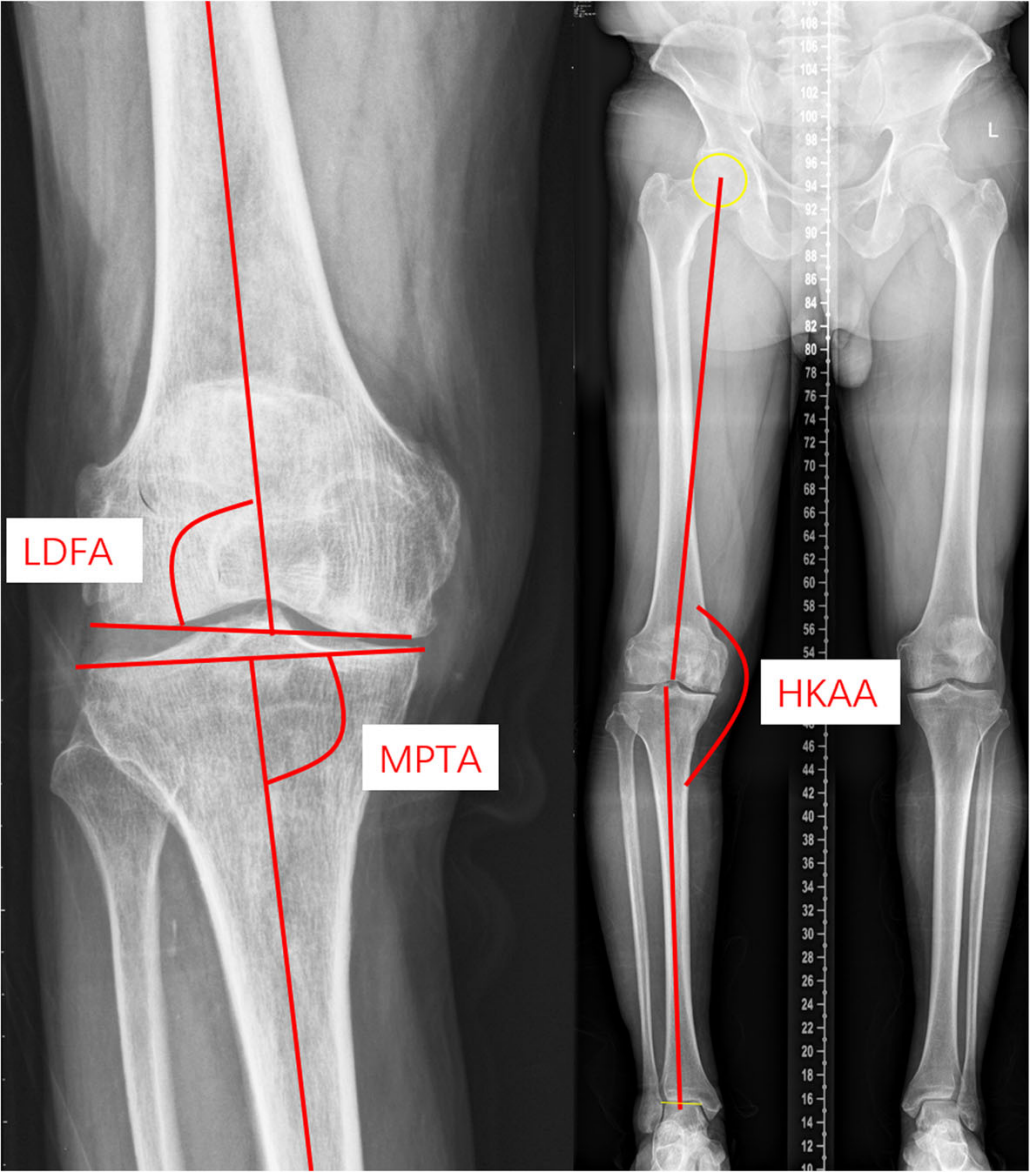
The patients were radiographically assessed using their preoperative weight-bearing X-rays. The overall limb alignment hip-knee-ankle angle (HKAA) was defined as the angle created by the hip center, center of the distal femoral notch, and center of the ankle talus. The lateral distal femoral angle (LDFA) was measured as the lateral angle between the distal femoral articular surface and the anatomical axis of the femur, while the medial proximal tibia angle (MPTA) was defined as the medial angle between the knee joint line of the tibia and axis line of the tibia

**Fig. 5:**
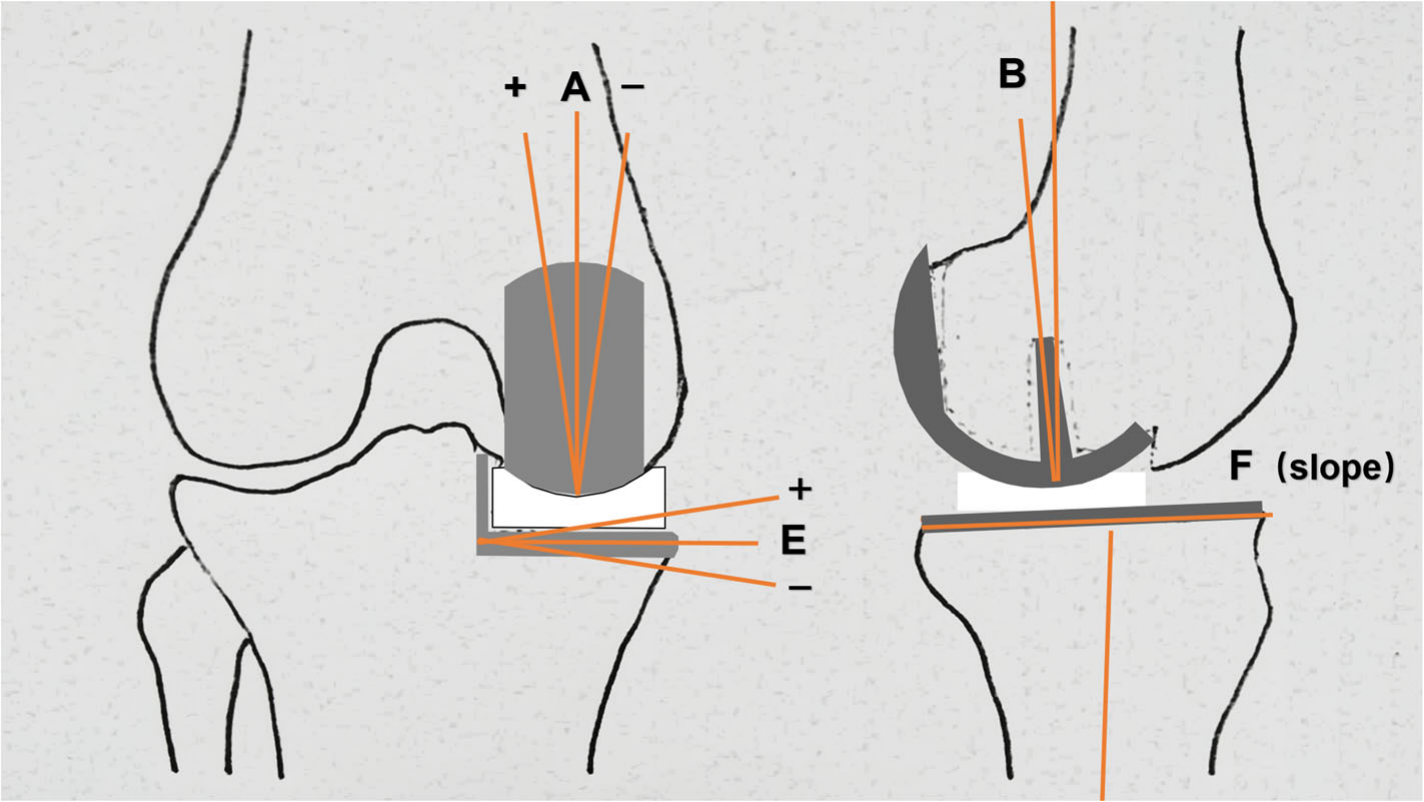
Diagrams showing the postoperative radiographic assessments of the component alignment and position: coronal angle of femoral component (femoral angle A), 10° varus to 10° valgus; sagittal angle of femoral component (femoral angle B), 15° flexion to 0° extension; coronal angle of tibial component (tibial angle E), 5° varus to 5° valgus; and posterior-inferior slope of tibial component (tibial angle F), 2° to 12°. Positive values represent varus and flexion alignment, and negative values represent valgus and extension alignment

Weight-bearing anteroposterior, lateral, and full-length weight-bearing radiographs were obtained at our institution both preoperatively and postoperatively. Care was taken to ensure that each patient stood with his or her patellae facing forward to minimize rotational variation among anteroposterior radiographs. Preoperative radiographic assessments included the hip-knee-ankle angle (HKAA), lateral distal femoral angle (LDFA), medial proximal tibial angle (MPTA), and tibial posterior slope. The postoperative implant position and alignment were assessed according to the guideline proposed by the Oxford group. Radiographic evaluation after UKA was defined as acceptable when the following angles were achieved: coronal angle of the femoral component (femoral angle A), 10° varus to 10° valgus; sagittal angle of femoral component (femoral angle B), 15° flexion to 0° extension; coronal angle of tibial component (tibial angle E), 5° varus to 5° valgus; and posterior-inferior slope of tibial component (tibial angle F), 2° to 12°. Angles beyond the acceptable limits were defined as outliers. For reliability assessment, radiological measurements were repeated after 3 weeks and the mean value was used for analysis (Figs. 4 and 5).

All data were analyzed using SPSS for Windows 17.0 (SPSS Inc., Chicago, IL, USA). The *χ*^2^ test and *t* test were used to determine statistically significant differences between groups. A *p* value of < 0.05 was considered statistically significant.

## Results

All patients were recruited at the final follow-up in August 2019. The mean follow-up for all patients was 39.76 ± 5.77 months and was at least 2 years (30–51 months). At baseline, the mean age at the time of the operation was 68.46 ± 9.07 years (range, 49–85 years), and the mean body mass index was 26.46 ± 3.36 kg/m^2^ (range, 18.3–35.2 kg/m^2^). In total, 46 UKAs were performed on the right knee and 54 on the left knee; 24 knees were male and 76 were female. The mean preoperative ROM was 122.88° ± 9.08° (range, 98°–134°), which improved to a mean of 125.36° ± 7.20° (range, 104°–140°) at the final follow-up (*t* = −3.404, *p* = 0.001). The mean HSS knee score increased from 59.23 ± 8.08 (range, 39–77) to 91.75 ± 5.21 (range, 75–99) at the final follow-up (t = −35.269*, p* = 0.000). The mean preoperative HKAA on weight-bearing radiographs was 173.41° ± 3.82° (range, 165.7°–179.8°), which was corrected to 177.29° ± 3.21° (range, 170.2°–186.3°) after UKA (*t* = −11.949*, p* = 0.000).

There were no differences in age, sex distribution, body mass index, follow-up, preoperative HSS score, ROM, or preoperative LDFA, MPTA, or tibial posterior slope between the two groups (Table 1). The mean length of time between surgery and the final follow-up was 39.34 ± 5.83 and 40.17 ± 5.74 months, respectively (*p =* 0.429). There were no differences in the postoperative HSS score (91.42 ± 5.21 and 92.18 ± 4.76; *p =* 0.448). The mean postoperative ROM in the modified group and conventional group was 125.96° ± 8.22° and 124.96° ± 5.70°, respectively (*p =* 0.484). There was also no difference in the postoperative alignment HKAA between the two groups (177.54° ± 3.35° and 177.05° ± 3.09°, *p =* 0.450). The drop in hemoglobin after 3 days in the modified group was only 12.34 ± 4.98 g/L, which was significantly less than that in the conventional group (*p =* 0.001). Correspondingly, the mean hemoglobin level in the modified group was higher than that in the conventional group. Moreover, the extramedullary technique decreased the operating time without the use of an intramedullary process. The operating times were 54.78 ± 7.95 and 59.14 ± 10.91 minutes, respectively (*p =* 0.025) (Table 1).

**Table 1:** Clinical outcomes in the modified group and the conventional group

	Group A—modified group(mean ± SD(range))	Group B—conventional group(mean ± SD(range))	*t*/*χ*2 value	*p* value
Number (knees)	50	50		
Sex (male/female)	11/39	13/37	0.219	0.640
Side (right/left)	25/25	21/29	0.644	0.422
Age (years)	69.14 ± 9.06(51~85)	67.78 ± 9.12(49~85)	0.748	0.456
BMI (kg/m^2^)	26.34 ± 3.90(18.3~35.2)	26.58 ± 2.76(20.8~31.1)	− 0.350	0.727
Mean follow-up (months)	39.34 ± 5.83 (30~51 )	40.17 ± 5.74(30~51)	− 0.794	0.429
Operating time (minutes)	54.78 ± 7.95(38~78)	59.14 ± 10.91(40~88)	− 2.284	0.025
Preoperative HSS score	58.60 ± 7.86(39~77)	59.86 ± 8.33(43~77)	− 0.778	0.438
Postoperative HSS score	91.42 ± 5.21(75~98)	92.18 ± 4.76(76~99)	− 0.761	0.448
Preoperative range of motion (degrees)	121.16 ± 9.76(98~132)	124.59 ± 8.07(108~134)	− 1.915	0.058
Postoperative range of motion (degrees)	125.96 ± 8.22(104~139)	124.96 ± 5.70(113~140)	0.703	0.484
Preoperative Hemoglobin (g/L)	134.12 ± 11.25(108~180)	134.00 ± 12.58(108~180)	0.050	0.960
Postoperative Hemoglobin (g/L)	121.78 ± 10.72(100~158)	117.26 ± 9.42(90~142)	2.240	0.027
Hemoglobin drop (g/L)	12.34 ± 4.98(4~25)	16.74 ± 7.54(5~38)	− 3.444	0.001

*Abbreviations*: *HSS* Hospital for Special Surgery, *ROM* range of motion

In the conventional group, one patient underwent revision to TKA after 6 months as a result of a tibial plateau fracture sustained in a major trauma. There were no clinical symptoms of implant failure or radiographic signs of loosening before the accident. One patient in each group reported continuing unexplained knee pain. No serious adverse events related to the operation, such as death, fat embolism, pulmonary embolism, periprosthetic joint infection, loosening, arthritis progression, or cardiocerebral vascular incidents, occurred in either group.

According to the guideline proposed in the surgical manual of the Oxford group, postoperative radiographic assessments showed that the accuracy of the implant position and alignment was comparable between the two groups (*p =* 0.678). Clinically acceptable implant alignment and position were achieved in 92% of patients in the modified group and 96% of patients in the conventional group. In the modified group, four UKAs were outliers of the acceptable limits: one femoral component tilted in the coronal plane with a postoperative femoral angle A of > 10°, one tilted with a femoral angle B of > 15°, one tibial component tilted in the coronal plane with a tibial angle E of > 5°, and one tilted with a tibial angle F of < 2°. In the conventional group, one femoral component tilted in the coronal plane with a postoperative femoral angle A of > 10° and one tilted with a femoral angle B of > 15°. There were no signs of implant failure at the final follow-up among these six UKAs. No significant differences were found in the mean postoperative femoral angle A, femoral angle B, tibial angle E, or tibial angle F between the two study groups (Table 2).

**Table 2:** Radiographic evaluation in the modified group and the conventional group

	Group A—modified group(mean ± SD(range))	Group B—conventional group(mean ± SD(range))	T value	*p* value
Preoperative HKAA (degrees)	173.27 ± 3.51(166.0~179.5)	173.55 ± 4.14(165.7~179.8)	− 0.362	0.718
Preoperative LDFA (degrees)	82.13 ± 2.57(77.1~88.5)	82.35 ± 2.71(75.3~88.9)	− 0.409	0.684
Preoperative MPTA (degrees)	85.35 ± 3.21(76.7~90.8)	85.79 ± 3.28(79.4~92.5)	− 0.670	0.505
Preoperative tibial posterior slope (degrees)	7.91 ± 3.56(0.2~15.8)	8.26 ± 3.61(− 0.1~16.3)	− 0.488	0.626
Postoperative HKAA (degrees)	177.54 ± 3.35(170.2~183.7)	177.05 ± 3.09(170.5~186.3)	0.758	0.450
Postoperative femoral angle A (degrees)	1.80 ± 3.46(− 4.9~13.0)	2.35 ± 2.35(− 2.9~11.1)	− 0.921	0.360
Postoperative femoral angle B (degrees)	5.31 ± 4.16(0.0~17.3)	4.66 ± 3.23(0.7~18.9)	0.870	0.387
Postoperative tibial angle E (degrees)	0.83 ± 2.09(− 3.5~8.9)	1.07 ± 1.75(− 2.4~4.8)	− 0.627	0.532
Postoperative tibial angle F (degrees)	6.87 ± 2.20(1.1~11.7)	6.36 ± 2.05(2.8~11.2)	1.203	0.232

*Abbreviations*: *HKAA* hip-knee-ankle angle, *LDFA* lateral distal femoral angle, *MPTA* medial proximal tibia angle

## Discussion

This is the first report of mobile bearing UKA using an extramedullary femoral guide technique. The most important finding of the present study was that the extramedullary technique for guiding femoral bone preparation based on the tibial cut first technique and overall alignment was as reliable and accurate as the conventional technique. Additionally, the extramedullary technique had a shorter operating time, less blood loss, and more rapid recovery without intramedullary interruption.

UKA has gained popularity worldwide since the introduction of this minimally invasive surgical technique by Repicci and Eberle [27]. However, mobile UKA is mostly regarded as a technical challenge because it is difficult to accurately implant the prosthetic components. In clinical practice, we have found that the intramedullary technique is complicated and not always accurate. Kim et al. [14] reported that among 124 Oxford phase 3 UKAs in 104 patients, 13% cases did not gain an acceptable postoperative mechanical axis. Alignment errors may have adverse effects on the wear rate of the mobile bearing, changes in knee kinematics, and implant loosening or failure [28]. Malpositioning may also increase the risk of bearing dislocation in mobile UKA, which highlights the importance of alignment accuracy [9, 10]. On radiographic evaluation, tibial prosthetic component alignment was usually good, and alignment as it was visually aligned and easily performed. However, maintaining accuracy on the femoral side may be difficult with the intramedullary technique, especially in the coronal plane. Intramedullary rod interference with the medial cortex during insertion may prevent further insertion or cause alignment errors. Therefore, we modified the femoral guide technique and used the tibial cut plane and overall alignment as references for femoral bone preparation without intramedullary interruption.

The advantage of the extramedullary technique using the tibial cut plane and overall alignment to guide the femoral bone cut is that it is easily performed and visually aligned in the coronal plane. The line drawn on the femur surface perpendicular to the tibial cut surface conforms to the limb alignment and femoral drill. In the sagittal plane, it is not difficult to achieve good alignment when the bottom of the femoral drill guide sits flatly on the tibial cut surface with the knee in 90° of flexion. Most importantly, because postoperative lower extremity alignment is crucial to the outcome of knee arthroplasty, varus deformity correction is an important concern during the operation. Alignment in UKA is determined by femoral and tibial bone resection without soft tissue release. The herein-described technique modifies the femoral preparation reference to the overall alignment in order to restore the natural alignment. In patients with anteromedial knee osteoarthritis, 1 to 2 mm of the distal femoral cartilage is usually lost and the posterior femoral cartilage is intact in flexion. Therefore, the extension gap is usually 1 to 2 mm larger than the flexion gap after the first tibial cut. A 1- to 2-mm larger gap gauge (compared with flexion) is then inserted into the joint space to correct the varus deformity to neutral limb alignment and maintain the natural tension of the ligament in extension. With the limb aligned in extension, the deformity may be passively corrected, and good alignment can be achieved.

The other advantage of this novel technique in UKA is that it eliminates the need for femoral intramedullary canal violation and reduces associated risks such as marrow emboli, postoperative hypoxia, and intraoperative fractures. Blood loss from the medullary canal is also significantly reduced in this novel technique. In the present study, the drop in hemoglobin after 3 days in the extramedullary technique group was only 12.34 g/L, which was significantly less than that in the conventional group (16.74 g/L). Moreover, it saved time without an intramedullary process. In this study, the operating times in the two groups were 54.78 and 59.14 minutes, respectively (*p =* 0.025). Most importantly, the study showed that the extramedullary technique using the tibial cut first technique and overall alignment to perform the distal femoral bone cut in UKA was as accurate as the conventional technique. Clinically acceptable implant alignment and positioning were achieved in 92% of patients in the extramedullary group and 96% of patients in the conventional group. No significant differences were found in the postoperative coronal and sagittal angles between the two groups. This reliable novel technique also produced clinical results comparable with those of the conventional technique. There were no differences in the postoperative HSS score or ROM between the two groups.

Patient outcomes depend on a meticulous surgical technique. The surgeon must remember that the proximal tibial cut in the coronal plane is the basic requirement for surgical success. When using the extramedullary technique in Oxford phase 3 UKA, the femoral drill guide should be visually aligned vertical to the tibial cut plane, in the middle of the condyle and parallel to the femoral reference line. The femoral drill guide is fixed with the hands, inducing a factor of uncertainty in the positioning of the femoral drill guide, which may result in an uncertain final position of the femoral component. To solve this problem, we usually check the alignment many times and confirm that no error has been made before femoral drilling. As the extramedullary technique mainly depends on the accurate tibial cut and overall alignment, we do not recommend the technique to surgeons without high volume experiences. Low volume is associated with an increased risk of revision, even using the conventional technique in UKA [29, 30]. In the study, the series were performed by an experienced surgeon who had performed over 1000 UKAs and had extreme confidence with this procedure; therefore, the result was encouraging. If a surgeon is not experienced with this process and cannot perform an accurate tibial cut, we do not recommend the extramedullary technique because an accurate tibial cut is the basic requirement for this novel technique.

This study has several limitations. First, it was limited to only the early postoperative results and complications. Further studies are required, including subsequent clinical results and revision reports. Second, all operations were performed by a surgeon experienced in UKA, and the results may differ in other scenarios. Third, this study was only performed within the Chinese population. Whether the method is also applicable to Western populations remains unclear.

## Conclusion

The clinical relevance of this study is that the extramedullary technique using the tibial cut first technique and overall alignment to guide femoral bone preparation in UKA was shown to be reliable and at least as accurate as the conventional technique. It has many advantages, such as a shorter operating time, less blood loss, less intramedullary interruption, and more rapid recovery.

## Data Availability

The datasets supporting the conclusions of this article are included within the article. The raw data underlying the conclusions made in this study can be obtained from the first author upon request.

## Abbreviations

HKAA: Hip-knee-ankle angle
HSS: Hospital for Special Surgery
LDFA: Lateral distal femoral angle
MPTA: Medial proximal tibia angle
ROM: Range of motion
TKA: Total knee arthroplasty
UKA: Unicompartmental knee arthroplasty
